# Rotavirus Prevalence, Genetic Diversity, and Co-Infections During the 2023–2024 Cholera Outbreak in Zambia: Insights from Multi-Pathogen Diagnostics

**DOI:** 10.3390/v18050508

**Published:** 2026-04-29

**Authors:** Adriace Chauwa, Samuel Bosomprah, Bernard Phiri, Natasha M. Laban, Dhvani H. Kuntawala, Dennis Ngosa, Harriet Ng’ombe, Fraser Liswaniso, Chaluma C. Luchen, Mutinta Muchimba, Innocent Mwape, Bertha T. Nzangwa, Sekayi F. Tigere, Kennedy Chibesa, Suwilanji Silwamba, Michelo Simuyandi, Nyuma Mbewe, Roma Chilengi, Caroline Chisenga

**Affiliations:** 1 Centre for Infectious Disease Research in Zambia, P.O. Box 34681, Lusaka 10101, Zambia; adriace.chauwa@cidrz.org (A.C.); samuel.bosomprah@cidrz.org (S.B.); phiribernard2013@gmail.com (B.P.); natasha.laban@cidrz.org (N.M.L.); dhvani.kuntawala@cidrz.org (D.H.K.); dennis.ngosa@cidrz.org (D.N.); harriet.ngombe@cidrz.org (H.N.); fraser.liswaniso@cidrz.org (F.L.); chaluma.luchen@cidrz.org (C.C.L.); mutinta.muchimba@cidrz.org (M.M.); innokatebe@gmail.com (I.M.); bertha.nzangwa@cidrz.org (B.T.N.); sekayi.tigere@cidrz.org (S.F.T.); kennedy.chibesa@cidrz.org (K.C.); suwilanji.silwamba@cidrz.org (S.S.); michelo.simuyandi@cidrz.org (M.S.); chilengir@yahoo.com (R.C.); 2 Department of Biostatistics, School of Public Health, University of Ghana, Accra P.O. Box LG13, Ghana; 3 Centre for Epidemic Response and Innovation, Stellenbosch University, Private Bag X1, Stellenbosch 7599, South Africa; 4 Department of Global Health, Amsterdam Institute for Global Health and Development, 1105 AZ Amsterdam, The Netherlands; 5 Zambia National Public Health Institute Reference Laboratory, Lusaka 10101, Zambia; nymbewe@gmail.com; 6 Parasites and Microbes Programme, Wellcome Sanger Institute, Cambridge CB10 1SA, UK

**Keywords:** diarrhoea, genotype diversity, surveillance, viral gastroenteritis

## Abstract

During Zambia’s 2023–2024 cholera outbreak, reliance on single-pathogen diagnostics risked overlooking co-circulating enteric pathogens. This study estimated the prevalence of rotavirus and described co-detected enteropathogens and rotavirus genotypes among patients admitted with suspected cholera. A sub-analysis was conducted on diarrhoeal stool specimens collected from patients who met the syndromic suspected cholera case definition. Samples were tested using the Bosphore^®^ Gastroenteritis Panel v2, a multiplex PCR enteric panel, to detect rotavirus and other gastrointestinal pathogens. Rotavirus-positive specimens with sufficient viral load were further genotyped by RT-PCR targeting of the VP7 and VP4 genes. Among 319 suspected cholera admissions, rotavirus was detected in 18 patients (5.6%; 95% CI 3.4–8.8%), predominantly in children aged <5 years (27.8%, 5/18) and 6–17 years (27.8%, 5/18). Co-infection was common, with 17/18 (94.4%) of rotavirus-positive samples showing co-infection with at least one additional enteric pathogen, most frequently *Campylobacter.* Genotyping was successful in five samples and revealed heterogenous circulating strains, including G1P[8], G2P[4], G3P[6], G12P[6], and G1P[6]. Rotavirus accounted for a modest proportion of suspected cholera admissions and was frequently detected in mixed enteric infections, underscoring the value of multi-pathogen diagnostics and continued molecular surveillance during outbreak response.

## 1. Introduction

Diarrhoeal diseases remain one of the leading causes of preventable morbidity and mortality worldwide, disproportionately affecting young children in low-resource settings. Each year, these illnesses are responsible for around 1.17 million deaths globally, with the WHO African region alone recording approximately 515,000 diarrhoeal deaths in 2020 [1]. National statistics in Zambia show that diarrhoea is among the top killers of children under five, causing an estimated 15,000 child deaths annually [2]. During declared cholera outbreaks, the singular diagnostic focus on *Vibrio cholerae* (*V. cholerae*) can obscure the true aetiology of diarrhoeal disease, particularly in children. This narrow approach misses co-circulating pathogens like rotavirus, limiting a comprehensive understanding of the disease burden and hindering targeted public health action.

Cholera’s acute watery diarrhoea is clinically indistinguishable from that caused by other pathogens like rotavirus and *Escherichia coli* (*E. coli*) [3]. The diagnostic overlap is often witnessed in outbreak settings; for example, in a cholera-endemic region of the Democratic Republic of Congo, just 38% of suspected cholera patients were PCR-positive for *V. cholerae*, while enterotoxigenic *E. coli* and Cryptosporidium were identified in 36% and 28% of cases, respectively [4]. Similarly, during a large diarrhoeal outbreak in Bangladesh, rotavirus emerged as the leading cause of acute diarrhoea among children under five (26% of cases), surpassing cholera in that age group [5]. Treating such non-cholera cases as cholera results in unnecessary antibiotic use that provides no benefit against viruses and instead fuels AMR [6], while this diagnostic oversight would miss an opportunity to document rotavirus genotypes, data which is crucial for evaluating vaccine effectiveness and efficacy. Therefore, the importance of detecting RV in outbreak contexts extends beyond case attribution.

Implementing multi-pathogen diagnostic panels during outbreaks yields two vital streams of data: molecular epidemiological intelligence on circulating strains and evidence of co-infection patterns with other enteric agents [7,8]. Rotavirus surveillance is particularly critical in countries implementing live attenuated vaccines, where ongoing viral evolution, reassortment, and genotype replacement may influence vaccine performance [9,10,11]. In this context, understanding rotavirus genetic diversity during large diarrhoeal outbreaks provides essential data to inform immunisation programmes and long-term disease control strategies. Zambia’s recent transition from Rotarix^®^ to Rotavac^®^—both live attenuated oral vaccines but derived from different strain formulations, underscores the need for continued surveillance to monitor circulating genotypes and detect shifts that may have implications for vaccine effectiveness [12]. Within this evolving immunisation landscape, systematic genomic monitoring enables timely detection of strain variation and supports evidence-based evaluation of vaccine performance.

The 2023–2024 cholera outbreak in Zambia provided a unique opportunity to leverage multi-pathogen diagnostics to assess rotavirus epidemiology beyond routine surveillance frameworks. In this study, we investigated the prevalence of rotavirus among clinically suspected cholera cases, characterised the genetic diversity of circulating rotavirus strains, and examined patterns of co-infection with other enteric pathogens.

## 2. Methods

Study Design: This laboratory-based analysis was conducted using specimens collected during a broader multi-pathogen investigation conducted during the 2023–2024 cholera outbreak, the primary findings of which were published in 2025 [13]. Cholera testing in the parent investigation included real-time PCR targeting the ctxA gene as previously described. Rapid diagnostic testing (RDT) was performed in accordance with outbreak triage procedures at selected centres but was not uniformly applied to all admissions. Clinical management decisions were based on case definitions used during the outbreak response. The present analysis did not re-examine cholera mono-infections but focused specifically on detection and characterisation of rotavirus of among participants admitted under suspected cholera case definitions during the outbreak.

Participants: A total of 319 patients from five cholera treatment centres across Lusaka and surrounding districts who presented with acute watery diarrhoea and met the clinical case definition for cholera were included in the analysis.

Stool sample processing: Stool specimens were processed as previously described [13]. Briefly, approximately 150 mg of stool was homogenised in SK 38 bead-beating tubes containing easyMAG^®^ Lysis Buffer (bioMérieux S.A., Marcy l’Etoile, France). The resulting homogenate was then centrifuged at 14,000 RPM for 2 min, and 200 μL of the supernatant was utilised for subsequent nucleic acid extraction using the Qiagen MinElute Kit (Qiagen, Hilden, Germany) according to the manufacturer’s instructions. Eluted nucleic acid was stored at −80 °C until analysis.

Purified nucleic acids were screened using the Bosphore^®^ Gastroenteritis Panel v2 (Anatolia Geneworks, Istanbul, Turkey), a multiplex real-time quantitative PCR assay targeting 11 enteric pathogens, including astrovirus, rotavirus, norovirus G1 and GII, adenovirus, while the bacterial pathogens comprise *Clostridium difficile*, *Campylobacter* spp., *Salmonella* spp., Enteroinvasive *E. coli* (EIEC) and *Shigella* spp., verotoxigenic *E. coli* (VTEC) and *Yersinia enterocolitica*. According to the manufacturer, the Bosphore^®^ Gastroenteritis Panel v2 has reported specificity exceeding 98% [7]. Nevertheless, at low pathogen prevalence, a small number of false-positive results may be expected. Interpretation was therefore based on manufacturer-defined amplification thresholds and review of amplification curves. The potential for false positives is acknowledged as a limitation. Reactions were run in a 25 µL reaction volume according to the manufacturer’s instructions, and run on the Real-time PCR Applied Biosystem Quantstudio 5 qPCR platform (Thermo Fisher Scientific, Waltham, MS, USA). Pathogen-specific detection was based on fluorescence signal threshold cycles (*Ct*), with *Ct* < 35 considered positive in accordance with manufacturer’s recommendations and prior validation studies [7]. Each run included internal amplification controls, positive controls, and no-template controls to monitor assay performance and contamination. Amplification curves were reviewed manually to confirm exponential signal patterns.

Rotavirus Genotyping: Rotavirus-positive samples with *Ct* ≤ 30 were selected for genotyping to maximise amplification success. In addition to extraction and internal amplification controls, amplification curves were reviewed to confirm exponential signal characteristics, and results near the positivity threshold were interpreted cautiously, particularly in the absence of quantitative viral load assessment. Of the 18 positive samples, 5 met this threshold and yielded successful VP7/VP4 amplicons. The G(VP7) and P(VP4) genotypes were determined using previously described semi-nested RT-PCR protocols employing Gouvea primers and Gentsch primer sets [14,15]. The RT-PCR assay was performed using the SuperScript^TM^ III One-Step RT-PCR System with Platinum^®^ Taq DNA Polymerase (Invitrogen, Carlsbad, CA, USA), under the following cycling conditions: reverse transcription at 50 °C for 30 min, initial denaturation at 94 °C for 2 min, followed by 35 cycles of 94 °C for 30 s, 42 °C for 30 s, and 68 °C for 1 min, with a final extension at 68 °C for 5 min. Amplicons were resolved by agarose gel electrophoresis and purified using a QIAquick PCR Purification Kit (Qiagen, Hilden, Germany).

## 3. Statistical Analysis

Demographic characteristics were summarised using descriptive statistics, with categorical variables presented as frequencies (percentages) and continuous variables as medians and interquartile intervals (IQIs), as per published guidelines [16]. Exact binomial 95% confidence intervals were calculated for prevalence estimates. Rotavirus positivity was reported as an overall proportion. Frequencies of rotavirus-positive participants were visualised across predefined age categories. Rotavirus genotypes were described, and their distribution was examined in relation to co-detected enteric pathogens using a genotype–pathogen co-occurrence plot. Participants were excluded only if multiplex PCR results were unavailable, or if demographic data were entirely missing. Demographic variables with missing values were retained in the dataset and explicitly reported as missing categories in descriptive analyses. All analyses were conducted using available-case denominators without imputation in STATA 18 (StataCorp, College Station, TX, USA).

## 4. Ethics Statement

Ethical approval for the parent study was obtained from the University of Zambia Biomedical Research Ethics Committee (UNZABREC; Ref: 001-02-23) and the National Health Research Authority (NHRA). Written informed consent was obtained from all adult participants or from the parents/legal guardians of children prior to their enrolment in the parent study. This sub-analysis on rotavirus data constitutes a secondary analysis within the scope of the original approved protocol. All data were de-identified and stored securely to ensure participant confidentiality. The datasets analyzed in this study are available from the corresponding author upon reasonable request, subject to institutional approval. Access is governed by the CIDRZ Ethics and Compliance Committee; therefore, formal inquiries should be directed to the Head of Research Operations (Hope Chinganya) at hope.chinganya@cidrz.org. All procedures were performed in accordance with the ethical standards of the responsible committees and with the Helsinki Declaration.

## 5. Results

### 5.1. Tracing of Study Participants

**Figure 1:**
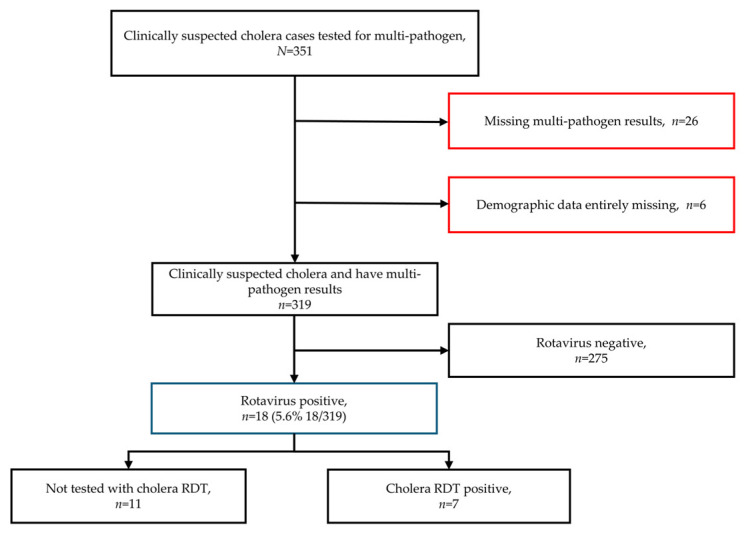
Study flow chart. Abbreviations: *N*, number of samples; *n*, number of samples in a given category; RDT, rapid diagnostic test; %, percentage. Red boxes indicate participants excluded from the final analytical dataset, while the blue box highlights the final sample used for rotavirus prevalence estimation.

The study workflow began with 351 patients who presented at the 5 cholera admission centres with acute watery diarrhoea meeting the clinical case definition for suspected cholera (Figure 1). After the exclusion of 32 individuals with missing demographic data and multi-pathogen results, 319 patients remained for analysis. Out of 319 participants, 18 individuals tested positive for rotavirus. Documented cholera rapid diagnostic test (RDT) results were available for 7 of the 18 rotavirus-positive individuals. The remaining 11 participants were not tested using RDT, likely reflecting operational constraints during emergency outbreak triage when diagnostic testing may not be systematically performed for all patients.

### 5.2. Descriptive Statistics of Study Participants

A summary of participant background characteristics is presented in Table 1. The median age was 24, with an interquartile range of 12 to 38. Specimen were collected from five health facilities; the majority came from Matero (37%), whereas the Levy and Chipata districts contributed 9 and 8%, respectively. Male participants represented 44% of the study population, females 31%, and sex data was missing for a notable 25%. Most participants were HIV negative (89%) and had missing data on cholera vaccination status (88%).

### 5.3. Prevalence of Rotavirus Infection Among Clinically Suspected Cholera Cases

Among 319 patients admitted to five cholera treatment centres with clinically suspected cholera and multi-pathogen results, rotavirus was detected in 18 cases, corresponding to a prevalence of 5.6% (18/319; 95% confidence interval: 3.4–8.8%).

### 5.4. Rotavirus Co-Infections with Other Enteric Pathogens

Analysis of pathogen co-detection patterns among 18 rotavirus-positive samples revealed that rotavirus was almost exclusively found in mixed infections. Only one case (5.6%, 1/18) involved a single rotavirus infection, while the majority of detections (94.4%, 17/18) involved at least one additional enteric pathogen. Dual-pathogen infections were the most common profile (38.9%, 7/18), followed by triple-pathogen combinations (22.2%, 4/18). We also identified more complex infections involving four (11.1%, 2/18), five (5.6%, 1/18), and six (16.7%, 3/18) pathogens. *Campylobacter* was the most prevalent bacterial co-pathogen, appearing alongside rotavirus in all multi-pathogen categories and frequently co-occurring with viral pathogens such as norovirus GI/GII and adenovirus. EIEC/*Shigella* and *Salmonella* were sporadically co-detected.

### 5.5. Rotavirus Genotypes

#### Rotavirus Genotypes and Age-Distribution

**Figure 2:**
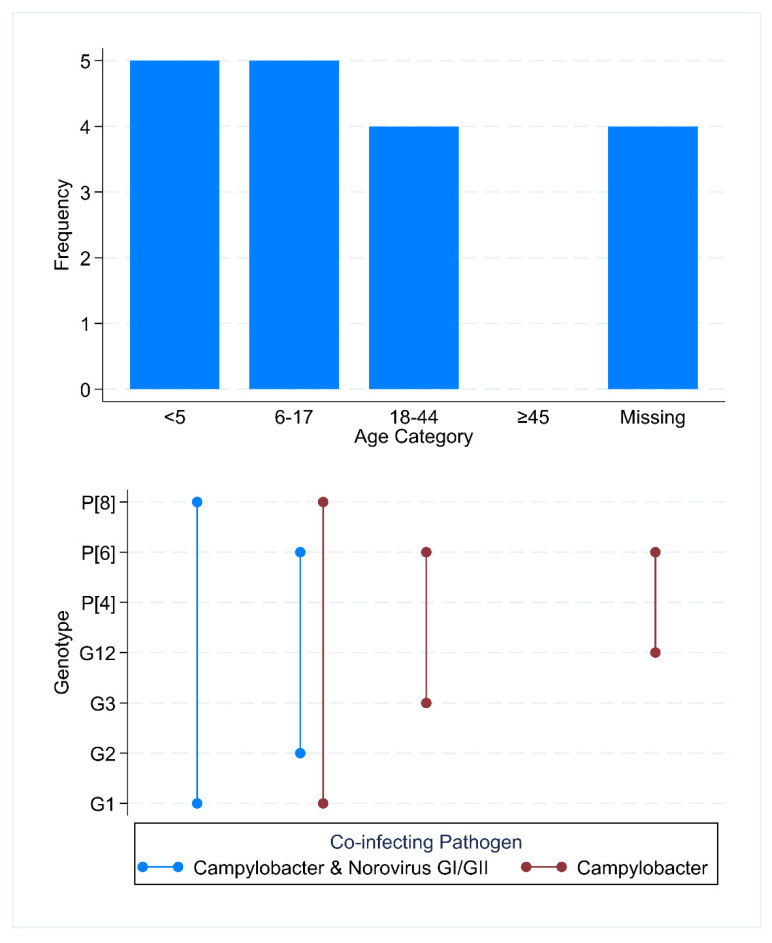
Distribution of rotavirus-positive samples across age categories and rotavirus co-detected pathogens. (**Top panel**): Age distribution of rotavirus-positive participants (*n* = 18), with age categories expressed in years. The ≥45 age group contained zero cases. “Missing” indicates unavailable age data. (**Bottom panel**): Rotavirus genotypes among successfully genotyped samples (*n* = 5), stratified by co-detecting pathogen(s). Blue markers represent *Campylobacter* and Norovirus GI/GII co-detections; red markers represent Campylobacter co-detection only.

Among rotavirus-positive cases, the highest proportion occurred in children aged <5 years (27.8%, 5/18) and 6–17 years (27.8%, 5/18). Five rotavirus samples met the genotyping threshold, and all showed evidence of co-infecting enteric pathogens. The identified strains demonstrated significant diversity, including globally common strains (G1P[8], G2P[4]), regionally prevalent strains (G12P[6], G3P[6]), and a rare reassortant (G1P[6]). In all cases, rotavirus was co-detected with at least one additional enteric pathogen, most frequently *Campylobacter* either alone or in combination with norovirus GI/GII (Figure 2).

## 6. Discussion

In this study, rotavirus accounted for a small portion of the diarrhoeal cases during the 2023–2024 cholera outbreak in Zambia, and it was predominantly identified in the context of co-infection. Although cholera was the primary outbreak pathogen for hospitalisation, these findings demonstrate that other clinically relevant enteric viruses continued to circulate concurrently. The observed prevalence is consistent with reports from other outbreak and post-vaccine settings, where rotavirus detection persists despite reductions in overall disease burden following vaccine introduction [17,18,19,20,21]. The frequency of mixed infections highlights the complexity of diarrhoeal aetiology in outbreak contexts, where overlapping transmission routes and environmental exposure may facilitate concurrent pathogen circulation [22]. Furthermore, the genotype heterogeneity observed among the limited number of successfully typed samples suggests ongoing viral diversity in the post-vaccine era. This finding underscores the importance of continued molecular surveillance, as previous studies have demonstrated that genotype diversity and reassortment continue to shape rotavirus epidemiology in the post-vaccine era [23,24].

An important insight from our investigation is the clinical overlap between cholera and rotavirus illness and its consequences for case management. The profuse watery diarrhoea, vomiting, and rapid dehydration characteristics are clinically indistinguishable from severe rotavirus gastroenteritis [25,26]. In the current study, this phenotypic overlap likely led clinicians to initially suspect cholera in rotavirus-infected patients, illustrating how syndromic definitions can mask the true aetiologies during outbreaks. The practical implication is that, without multiplex diagnostics, viral diarrhoeas may be misclassified as cholera, resulting in inappropriate management [27,28]. Patients with unrecognised rotavirus infection might be admitted to cholera treatment centres and administered unnecessary antibiotics, which provide no benefit against viruses and instead contribute to antimicrobial resistance [13]. At the same time, misattribution hinders pathogen-specific surveillance, appropriate antimicrobial stewardship and the collection of rotavirus strain data critical for evaluating vaccine performance in the post-vaccine era [29,30,31]. These considerations strongly support the adoption of multi-pathogen testing in outbreak investigations, to ensure that co-circulating infections are correctly identified and treated, and that surveillance captures all major contributors to the outbreak’s morbidity.

One notable finding was the moderately high rate of co-infections among rotavirus-positive individuals, underscoring the complex aetiology of diarrhoeal outbreaks. Nearly 94% of rotavirus-positive cases harboured at least one additional enteric pathogen—most often *Campylobacter* (either alone or in combination with norovirus GI/GII). Dual infections were common, and a sizable fraction of cases had three concurrent pathogens, suggesting that clinical disease may have involved multiple overlapping infections rather than a single-agent. These co-infection of *Campylobacter* as a recurring partner to rotavirus across different age groups, suggests shared transmission pathways or environmental sources that facilitated their joint circulation. This finding mirrors observations from the broader outbreak investigation in Zambia where 80% of suspected cholera cases actually involved mixed infections, with *Campylobacter* and norovirus GI/GII frequently accompanying other pathogens [13]. Likewise, diarrhoeal outbreaks in other settings have shown that presumed cholera cases often harbour diverse pathogens such as *E. coli* and *Cryptosporidium* alongside (or instead of) *V. cholerae* [3,4,5]. These aetiologic complexities create challenges for treatment. For example, a patient co-infected with rotavirus (virus) and *Campylobacter* (bacterium) might require rehydration and careful use of antibiotics, whereas a misdiagnosis of “cholera only” could lead to suboptimal care. The frequent detection of multiple enteric pathogens reflects the high background burden of enteric infections, and underscores why an integrated diagnostic and surveillance strategy is critical in outbreak settings. Multiplex PCR platforms may provide valuable epidemiological insights when integrated into reference laboratory support during outbreaks, although their use in field triage settings may be limited by cost and infrastructure requirements. Additionally, multiplex PCR detects nucleic acid and does not distinguish between active infection, transient carriage, or residual shedding. Clinical correlation remains essential in attributing causality.

Genotyping analysis revealed a remarkable diversity of rotavirus strains circulating during the outbreak, including both vaccine-related and unusual genotypes. The data revealed classic human strains that are targeted by vaccines such as G1P[8] and G2P[4], co-circulating with less common types like G12P[6] and G3P[6], as well as the rare reassortant strain G1P[6] that is not typically seen in humans. Importantly, none of the rotavirus-positive patients in this study were co-infected with *V. cholerae*, indicating that rotavirus and cholera infections occurred in parallel rather than within the same individuals. The presence of multiple rotavirus genotypes in this context mildly suggests complex transmission dynamics at play. On one hand, the identification of G1P[8] and G2P[4], strains against which the Rotarix^®^ vaccine is designed to protect, alongside other genotypes could imply that routine vaccination was not fully interrupting rotavirus circulation during the outbreak. However, the small sample size precludes broader conclusions regarding genotype diversity or potential vaccine-era dynamics within the study population.

The appearance of unusual strains points to introduction from outside sources or virus evolution. Notably, the emergence of genotype G2P[4] among outbreak strains is consistent with reports from several African countries [32,33,34]. Further, detection of this strain underscores the importance of ongoing genomic surveillance to detect strains that may have implications for vaccine performance consistent with recommendations from previous studies [35,36,37]. Research elsewhere hypothesises that shifts in genotype distribution following vaccine introduction may reflect complex interactions between host immunity, viral fitness, and natural genotype fluctuation [33]. The detection of a reassortant G1P[8] strain in this study during an outbreak scenario aligns with previous post-vaccine reports, where such strains were implicated in break-through rotavirus gastroenteritis [29,38,39]. However, the present study was not designed to evaluate vaccine effectiveness or selective pressures, and therefore no inference can be made regarding vaccine-driven selection.

The detection of a G12P[6] in this cohort is notable, as this genotype combination has been previously associated with zoonotic reassortment events [35,40,41]. Genotype G12, particularly when paired with P[6], has been increasingly reported across sub-Saharan Africa and has, in some cases, been shown through whole-genome analyses to carry internal gene segments of porcine origin [18,35,41,42,43]. However, the present study did not include full genome characterisation and therefore the origin of the detected G12P[6] cannot be determined. Nonetheless, its identification aligns with patterns of genotype diversity reported in the region and underscores the dynamic nature of rotavirus evolution. Such reassortment events are epidemiologically important as they can generate novel rotavirus variants with unpredictable antigenicity or virulence, potentially undermining existing immunity in the human population. This highlights the need for a One Health approach in surveillance, as the health of human populations may be directly affected by rotavirus strains circulating in livestock and other animals.

The identification of a rare G1P[6] strain represents an uncommon genotype combination in the African context. While P[6] is typically detected in association with G12 or other strains linked to zoonotic or reassortant origins, its pairing with G1 is less commonly reported. Large post-vaccine genotype surveys from Nigeria and Nepal have document atypical genotype constellations; however, G1P[6] has remained relatively infrequent [34,40]. The presence of this combination during the Zambian outbreak could signify an uncommon reassortment events occurring in settings where multiple strains co-circulate, although the limited sample size precludes definitive conclusions regarding its origin. Similar patterns of emerging novel genotypes have been observed in other African countries [44,45,46], underscoring the dynamic nature of rotavirus evolution in the vaccine era. Continued genomic surveillance will be important to determine whether such uncommon genotype constellations represent isolated detections or broader shifts in circulating strain populations.

The findings of this study provide important insights for cholera outbreak response and enteric pathogen surveillance, challenging the traditional single-pathogen paradigm. Large multicentre studies of diarrhoeal disease, including the Global Enteric Multicentre Study (GEMS) and MAL-ED, have demonstrated that multiple pathogens frequently contribute to diarrhoeal episodes and that clinical features alone are insufficient for reliable aetiologic attribution [8,47,48]. In the present outbreak, rotavirus was detected in 5.6% of patients meeting the clinical case definition for cholera, reinforcing evidence that reliance solely on syndromic presentation or pathogen-specific rapid testing may mask the contribution of other enteric pathogens to overall disease burden [49,50].

While evidence from respiratory infections in high-income paediatric intensive care units suggests that viral PCR detection does not automatically reduce antibiotic prescribing [51], the application of multiplex PCR platforms for enteric pathogens presents a distinct opportunity. In outbreak settings, these panels have been shown to substantially increase pathogen detection compared to standard methods [52,53], and studies from high-burden settings confirm that enteric viruses contribute significantly to diarrhoeal disease, frequently as mixed infections [54]. By definitively identifying viral aetiologies that do not require antibiotic therapy, multiplex diagnostics may support antimicrobial stewardship efforts when integrated into clinical decision-making frameworks. However, the enhanced sensitivity of these assays necessitates careful interpretation, particularly in high-burden settings where asymptomatic carriage and low-level co-detections of enteric pathogens are common [7,55]. The notable frequency of mixed infections reported in a previous study further illustrates this challenge, as distinguishing the primary causative pathogen often requires quantitative thresholds and clinical correlation [54]. Together, these findings suggest that while broad-spectrum diagnostics can enrich aetiologic understanding during outbreaks, their effective integration into response frameworks must balance diagnostic sensitivity with interpretive rigour to optimise patient management and minimise unnecessary antibiotic use.

A key strength of this study is that it leveraged a broad multiplex PCR platform to identify rotavirus and a wide range of co-circulating enteropathogens, thereby providing an aetiologically richer picture than routine single-pathogen surveillance and enabling the description of non-random co-detection patterns. In addition, molecular characterisation of circulating strains, albeit in a subset, adds value by demonstrating genotype diversity, including uncommon and potentially zoonotic-associated types, relevant to vaccine-era monitoring and outbreak preparedness.

Important limitations should also be noted. The analysis reflects a convenience sample of treatment-centre admissions defined by syndromic criteria and therefore may not be generalisable to community diarrhoea or to milder presentations, and the small number of rotavirus-positive cases limited precision and reduced the power to detect associations based on demographic or clinical factors. Genotyping was possible for only a small fraction of positive samples, which constrains inference about population-level strain distribution. Given the high reported specificity of the multiplex platform (>98%), a small number of false-positive detections may occur, particularly at low prevalence. This should be considered when interpreting pathogen co-detections identified in this study. A further limitation relates to the substantial proportion of missing demographic information in the dataset, particularly for sex, age categories, and cholera vaccination status. Such missingness may influence interpretation of the apparent demographic distribution of cases reported in this study. In outbreak response settings, data collection often occurs under operational constraints, where clinical management and rapid triage take precedence over complete documentation. As a result, incomplete recording of demographic variables can occur, and the patterns observed in this analysis should therefore be interpreted with caution. Finally, key clinical and exposure variables (including rotavirus vaccination history, symptom severity, antibiotic use, WASH exposures, and complete HIV-related information) were incompletely captured during the emergency response, increasing the potential for residual confounding and limiting the interpretation of pathways linking pathogen detections to clinical outcomes.

## 7. Conclusions

Rotavirus contributed a modest but clinically meaningful proportion of admissions labelled as suspected cholera during Zambia’s 2023–2024 outbreak, and it was detected predominantly in children and adolescents and most often within mixed enteric infections. The frequent co-detection of pathogens such as *Campylobacter* and norovirus underscores the aetiologic complexity that can underlie cholera-like presentations and highlights the limitations of syndromic diagnosis during outbreaks. The diversity of rotavirus genotypes identified, including uncommon and potentially zoonotic-associated or reassortant strains, reinforces the importance of coupling routine case surveillance with molecular characterisation to track strain evolution and to inform vaccine-era monitoring, particularly as Zambia transitions between vaccine products. Together, these findings support a more integrated outbreak response that incorporates multi-pathogen diagnostics, judicious antimicrobial stewardship, and strengthened genomic surveillance within a One Health framework to improve clinical management and guide targeted public health action for diarrhoeal disease control.

## Figures and Tables

**Table 1:** Background characteristics of study population.

Characteristics	Total *N* = 319
	*n* (% of total)
Sex	
Male	141 (44.2)
Female	100 (31.3)
Missing	78 (24.5)
Age (years)	
Midian (IQR ^†^)	24 (12–38)
Age group (years)	
Infants & Young Children (<5)	31 (9.7)
Children/Adolescents (6–17)	44 (13.8)
Young Adults/Adults (18–44)	138 (43.3)
Older Adults (45+)	38 (11.9)
Missing age	68 (21.3)
Facility	
Chipata	26 (8.2)
George	74 (23.2)
Heroes	71 (22.3)
Levy	29 (9.1)
Matero	119 (37.3)
Vaccinated against cholera	
No	32 (10.0)
Yes	5 (1.6)
Missing	282 (88.4)
HIV Status	
Negative	283 (88.7)
Positive	30 (9.4)
Missing	6 (1.9)

Abbreviations: *N*: number of samples; %: Percentage; ^†^ IQR: Interquartile range.

## Data Availability

All data generated and analyzed during this study are included in the published manuscript. The data presented in this study are available upon reasonable request from the corresponding author. The CIDRZ Ethics and Compliance Committee is responsible for approving such request. To request data access, one must write to the Secretary to the Committee/Head of Research Operations, Hope Chinganya (hope.chinganya@cidrz.org). Dataset requests must include contact information, a research project title, a description of the proposed analysis, and the format in which it is expected to be provided. The requested data should only be used for the purposes related to the original research or study. The CIDRZ Ethics and Compliance Committee will normally review all data requests within 48–72 h (Monday–Friday) and provide notification if access has been granted or additional project information is needed before access can be granted.

## References

[1] Thystrup C., Majowicz S.E., Kitila D.B., Desta B.N., Fayemi O.E., Ayolabi C.I., Hugho E., Buys E.M., Akanni G.B., Machava N.E. (2024). Etiology-Specific Incidence and Mortality of Diarrheal Diseases in the African Region: A Systematic Review and Meta-Analysis. BMC Public Health.

[2] Hamooya B.M., Masenga S.K., Halwiindi H. (2020). Predictors of Diarrhea Episodes and Treatment-Seeking Behavior in under-Five Children: A Longitudinal Study from Rural Communities in Zambia. Pan Afr. Med. J..

[3] Wiens K.E., Xu H., Zou K., Mwaba J., Lessler J., Malembaka E.B., Demby M.N., Bwire G., Qadri F., Lee E.C. (2023). Estimating the Proportion of Clinically Suspected Cholera Cases That Are True Vibrio Cholerae Infections: A Systematic Review and Meta-Analysis. PLoS Med..

[4] Williams C. (2020). Prevalence and Diversity of Enteric Pathogens among Cholera Treatment Centre Patients with Acute Diarrhea in Uvira, Democratic Republic of Congo. BMC Infect. Dis..

[5] Hasan S.M.T., Das S., Faruque A.S.G., Khan A.I., Clemens J.D., Ahmed T. (2021). Taking Care of a Diarrhea Epidemic in an Urban Hospital in Bangladesh: Appraisal of Putative Causes, Presentation, Management, and Deaths Averted. PLoS Neglected Trop. Dis..

[6] Efunshile A.M., Ezeanosike O., Nwangwu C.C., König B., Jokelainen P., Robertson L.J. (2019). Apparent Overuse of Antibiotics in the Management of Watery Diarrhoea in Children in Abakaliki, Nigeria. BMC Infect. Dis..

[7] Liu J., Platts-Mills J.A., Juma J., Kabir F., Nkeze J., Okoi C., Operario D.J., Uddin J., Ahmed S., Alonso P.L. (2016). Use of Quantitative Molecular Diagnostic Methods to Identify Causes of Diarrhoea in Children: A Reanalysis of the GEMS Case-Control Study. Lancet.

[8] Platts-Mills J.A., Babji S., Bodhidatta L., Gratz J., Haque R., Havt A., McCormick B.J., McGrath M., Olortegui M.P., Samie A. (2015). Pathogen-Specific Burdens of Community Diarrhoea in Developing Countries: A Multisite Birth Cohort Study (MAL-ED). Lancet Glob. Health.

[9] Malakalinga J.J., Misinzo G., Msalya G.M., Kazwala R.R. (2019). Rotavirus Burden, Genetic Diversity and Impact of Vaccine in Children under Five in Tanzania. Pathogens.

[10] Hungerford D., Allen D.J., Nawaz S., Collins S., Ladhani S., Vivancos R., Iturriza-Gómara M. (2019). Impact of Rotavirus Vaccination on Rotavirus Genotype Distribution and Diversity in England, September 2006 to August 2016. Eurosurveillance.

[11] Lee B. (2021). Update on Rotavirus Vaccine Underperformance in Low- to Middle-Income Countries and next-Generation Vaccines. Hum. Vaccin. Immunother..

[12] Mpabalwani E.M., Sakala C., Kamiji E., Simwaka J., Soko J., Kabwe M., Chisanga A., Chisanga K., Sakala J., Kiulia N.M. (2025). Challenges and Lessons Learned during the Switching of Rotavirus Vaccine from Rotarix to Rotavac in Zambia. Vaccine.

[13] Kuntawala D.H., Bosomprah S., Phiri B., Ng’ombe H., Liswaniso F., Muchimba M., Silwamba S., Chibesa K., Nzangwa B.T., Luchen C.C. (2025). Prevalence and Patterns of Enteric Co-Infections Among Individuals Presenting with Cholera-like Diarrheal Disease During Seasonal Cholera Outbreaks. Pathogens.

[14] Gouvea V., Glass R.I., Woods P., Taniguchi K., Clark H.F., Forrester B., Fang Z.Y. (1990). Polymerase Chain Reaction Amplification and Typing of Rotavirus Nucleic Acid from Stool Specimens. J. Clin. Microbiol..

[15] Gentsch J.R., Glass R.I., Woods P., Gouvea V., Gorziglia M., Flores J., Das B.K., Bhan M.K. (1992). Identification of Group A Rotavirus Gene 4 Types by Polymerase Chain Reaction. J. Clin. Microbiol..

[16] Lang T.A., Altman D.G. (2015). Basic Statistical Reporting for Articles Published in Biomedical Journals: The “Statistical Analyses and Methods in the Published Literature” or the SAMPL Guidelines. Int. J. Nurs. Stud..

[17] Saha R., Lo M., De P., Deb A.K., Indwar P., Miyoshi S., Kitahara K., Oka T., Dutta S., Chawla-Sarkar M. (2025). Epidemiology of Viral Gastroenteritis in Children and Genetic Diversity of Rotavirus Strains in Kolkata, West Bengal after Introduction of Rotavirus Vaccine. Vaccine.

[18] Kabayiza J.-C., Nilsson S., Andersson M. (2023). Rotavirus Infections and Their Genotype Distribution in Rwanda before and after the Introduction of Rotavirus Vaccination. PLoS ONE.

[19] Badur S., Öztürk S., Pereira P., AbdelGhany M., Khalaf M., Lagoubi Y., Ozudogru O., Hanif K., Saha D. (2019). Systematic Review of the Rotavirus Infection Burden in the WHO-EMRO Region. Hum. Vaccines Immunother..

[20] Iturriza-Gómara M., Jere K.C., Hungerford D., Bar-Zeev N., Shioda K., Kanjerwa O., Houpt E.R., Operario D.J., Wachepa R., Pollock L. (2019). Etiology of Diarrhea Among Hospitalized Children in Blantyre, Malawi, Following Rotavirus Vaccine Introduction: A Case-Control Study. J. Infect. Dis..

[21] Chirinda P., Manjate F., Garrine M., Messa A., Nobela N., Vubil D., Nhampossa T., Acácio S., Bassat Q., Kotloff K.L. (2024). Detection of Enteric Viruses in Children under Five Years of Age before and after Rotavirus Vaccine Introduction in Manhiça District, Southern Mozambique, 2008–2019. Viruses.

[22] Li C., Xi L., Rao J., Xiang Y., Tang F., Wang X. (2025). Time-Series Analysis of Climatic Drivers of Pediatric Rotavirus and Adenovirus Infections in Post-Pandemic China. BMC Public Health.

[23] Vaselli N.M., Iturriza-Gómara M., Hungerford D., Members E. (2025). network Contribution of the EuroRotaNet Surveillance Network to Rotavirus Strain Surveillance in Europe. Eurosurveillance.

[24] Mwape I., Laban N.M., Chibesa K., Moono A., Silwamba S., Malisheni M.M., Chisenga C., Chauwa A., Simusika P., Phiri M. (2023). Characterization of Rotavirus Strains Responsible for Breakthrough Diarrheal Diseases among Zambian Children Using Whole Genome Sequencing. Vaccines.

[25] Parashar U.D., Nelson E.A.S., Kang G. (2013). Diagnosis, Management, and Prevention of Rotavirus Gastroenteritis in Children. BMJ.

[26] Bányai K., Estes M.K., Martella V., Parashar U.D. (2018). Viral Gastroenteritis. Lancet.

[27] Kowada K., Takeuchi K., Hirano E., Toho M., Sada K. (2018). Development of a Multiplex Real-Time PCR Assay for Detection of Human Enteric Viruses Other than Norovirus Using Samples Collected from Gastroenteritis Patients in Fukui Prefecture, Japan. J. Med. Virol..

[28] Wang J., Xu Z., Niu P., Zhang C., Zhang J., Guan L., Kan B., Duan Z., Ma X. (2014). A Two-Tube Multiplex Reverse Transcription PCR Assay for Simultaneous Detection of Viral and Bacterial Pathogens of Infectious Diarrhea. BioMed Res. Int..

[29] Amin A.B., Cates J.E., Liu Z., Wu J., Ali I., Rodriguez A., Panjwani J., Tate J.R., Lopman B.A., Parashar U.D. (2024). Rotavirus Genotypes in the Postvaccine Era: A Systematic Review and Meta-Analysis of Global, Regional, and Temporal Trends by Rotavirus Vaccine Introduction. J. Infect. Dis..

[30] Platts-Mills J.A., Amour C., Gratz J., Nshama R., Walongo T., Mujaga B., Maro A., McMurry T.L., Liu J., Mduma E. (2017). Impact of Rotavirus Vaccine Introduction and Postintroduction Etiology of Diarrhea Requiring Hospital Admission in Haydom, Tanzania, a Rural African Setting. Clin. Infect. Dis..

[31] Steele A.D., Groome M.J. (2020). Measuring Rotavirus Vaccine Impact in Sub-Saharan Africa. Clin. Infect. Dis..

[32] Makori T.O., Bargul J.L., Lambisia A.W., Mwanga M.J., Murunga N., De Laurent Z.R., Lewa C.S., Mutunga M., Kellam P., Cotten M. (2023). Genomic Epidemiology of the Rotavirus G2P[4] Strains in Coastal Kenya Pre- and Post-Rotavirus Vaccine Introduction, 2012–2018. Virus Evol..

[33] Mwangi P.N., Page N.A., Seheri M.L., Mphahlele M.J., Nadan S., Esona M.D., Kumwenda B., Kamng’ona A.W., Donato C.M., Steele D.A. (2022). Evolutionary Changes between Pre- and Post-Vaccine South African Group A G2P[4] Rotavirus Strains, 2003–2017. Microb. Genom..

[34] Adah M.I., Wade A., Taniguchi K. (2001). Molecular Epidemiology of Rotaviruses in Nigeria: Detection of Unusual Strains with G2P[6] and G8P[1] Specificities. J. Clin. Microbiol..

[35] Rakau K.G., Nyaga M.M., Gededzha M.P., Mwenda J.M., Mphahlele M.J., Seheri L.M., Steele A.D. (2021). Genetic Characterization of G12P[6] and G12P[8] Rotavirus Strains Collected in Six African Countries between 2010 and 2014. BMC Infect. Dis..

[36] Simwaka J.C., Mpabalwani E.M., Seheri M., Peenze I., Monze M., Belem M., Parashar U.D., Jacob M., Mphahlele J.M., Tate J.E. (2018). Diversity of Rotavirus Strains Circulating in Children under Five Years of Age Who Presented with Acute Gastroenteritis before and after Rotavirus Vaccine Introduction, University Teaching Hospital, Lusaka, Zambia, 2008–2015. Vaccine.

[37] Shrestha J., Shrestha S.K., Strand T.A., Dudman S., Dembinski J.L., Vikse R., Andreassen A.K. (2021). Diversity of Rotavirus Strains in Children; Results from a Community-Based Study in Nepal. Front. Med..

[38] Zeller M., Donato C., Trovão N.S., Cowley D., Heylen E., Donker N.C., McAllen J.K., Akopov A., Kirkness E.F., Lemey P. (2015). Genome-Wide Evolutionary Analyses of G1P[8] Strains Isolated Before and After Rotavirus Vaccine Introduction. Genome Biol. Evol..

[39] Jere K.C., Chaguza C., Bar-Zeev N., Lowe J., Peno C., Kumwenda B., Nakagomi O., Tate J.E., Parashar U.D., Heyderman R.S. (2018). Emergence of Double- and Triple-Gene Reassortant G1P[8] Rotaviruses Possessing a DS-1-Like Backbone after Rotavirus Vaccine Introduction in Malawi. J. Virol..

[40] Uchida R., Pandey B.D., Sherchand J.B., Ahmed K., Yokoo M., Nakagomi T., Cuevas L.E., Cunliffe N.A., Hart C.A., Nakagomi O. (2006). Molecular Epidemiology of Rotavirus Diarrhea among Children and Adults in Nepal: Detection of G12 Strains with P[6] or P[8] and a G11P[25] Strain. J. Clin. Microbiol..

[41] Mokoena F., Esona M.D., Seheri L.M., Nyaga M.M., Magagula N.B., Mukaratirwa A., Mulindwa A., Abebe A., Boula A., Tsolenyanu E. (2021). Whole Genome Analysis of African G12P[6] and G12P[8] Rotaviruses Provides Evidence of Porcine-Human Reassortment at NSP2, NSP3, and NSP4. Front. Microbiol..

[42] Azaran A., Makvandi M., Samarbafzadeh A., Neisi N., Hoseinzadeh M., Rasti M., Teymurirad M., Teimoori A., Varnaseri M., Makvandi K. (2016). Study on Rotavirus Infection and Its Genotyping in Children Below 5 Years in South West Iran. Iran. J. Pediatr..

[43] Nyaga M.M., Jere K.C., Esona M.D., Seheri M.L., Stucker K.M., Halpin R.A., Akopov A., Stockwell T.B., Peenze I., Diop A. (2015). Whole Genome Detection of Rotavirus Mixed Infections in Human, Porcine and Bovine Samples Co-Infected with Various Rotavirus Strains Collected from Sub-Saharan Africa. Infect. Genet. Evol..

[44] Gómez M.M., da Silva M.F.M., Zeller M., Heylen E., Matthijnssens J., Ichihara M.Y.T., Rose T.L., de Mello Volotão E., Leite J.P.G. (2013). Phylogenetic Analysis of G1P[6] Group A Rotavirus Strains Detected in Northeast Brazilian Children Fully Vaccinated with Rotarix^TM^. Infect. Genet. Evol..

[45] Jampanil N., Kumthip K., Maneekarn N., Khamrin P. (2023). Genetic Diversity of Rotaviruses Circulating in Pediatric Patients and Domestic Animals in Thailand. Trop. Med. Infect. Dis..

[46] Ghosh S., Urushibara N., Chawla-Sarkar M., Krishnan T., Kobayashi N. (2013). Whole Genomic Analyses of Asymptomatic Human G1P[6], G2P[6] and G3P[6] Rotavirus Strains Reveal Intergenogroup Reassortment Events and Genome Segments of Artiodactyl Origin. Infect. Genet. Evol..

[47] Kotloff K.L., Nataro J.P., Blackwelder W.C., Nasrin D., Farag T.H., Panchalingam S., Wu Y., Sow S.O., Sur D., Breiman R.F. (2013). Burden and Aetiology of Diarrhoeal Disease in Infants and Young Children in Developing Countries (the Global Enteric Multicenter Study, GEMS): A Prospective, Case-Control Study. Lancet.

[48] Lappan R., Jirapanjawat T., Williamson D.A., Lange S., Chown S.L., Greening C. (2022). Simultaneous Detection of Multiple Pathogens with the TaqMan Array Card. MethodsX.

[49] Axelrad J.E., Joelson A., Nobel Y., Whittier S., Lawlor G., Riddle M.S., Green P.H.R., Lebwohl B. (2018). The Distribution of Enteric Infections Utilizing Stool Microbial Polymerase Chain Reaction Testing in Clinical Practice. Dig. Dis. Sci..

[50] Wilber E., Baker J.M., Rebolledo P.A. (2021). Clinical Implications of Multiplex Pathogen Panels for the Diagnosis of Acute Viral Gastroenteritis. J. Clin. Microbiol..

[51] Hayotte A., Mariani-Kurkdjian P., Boizeau P., Dauger S., Riaud C., Lacarra B., Bourmaud A., Levy M. (2023). Viral Identification Using Multiplex Polymerase Chain Reaction Testing Does Not Reduce Antibiotic Prescribing in Paediatric Intensive Care Units. Microorganisms.

[52] Poritz M.A., Lingenfelter B., Tang Y.-W., Stratton C.W. (2018). Multiplex PCR for Detection and Identification of Microbial Pathogens. Advanced Techniques in Diagnostic Microbiology: Volume 2: Applications.

[53] Stockmann C., Pavia A.T., Graham B., Vaughn M., Crisp R., Poritz M.A., Thatcher S., Korgenski E.K., Barney T., Daly J. (2017). Detection of 23 Gastrointestinal Pathogens Among Children Who Present with Diarrhea. J. Pediatr. Infect. Dis. Soc..

[54] Tatte V.S., Gopalkrishna V. (2019). Detection of Different Enteric Viruses in Children with Diarrheal Disease: Evidence of the High Frequency of Mixed Infections. Access Microbiol..

[55] Zautner A.E., Groß U., Emele M.F., Hagen R.M., Frickmann H. (2017). More Pathogenicity or Just More Pathogens?—On the Interpretation Problem of Multiple Pathogen Detections with Diagnostic Multiplex Assays. Front. Microbiol..

